# Difficulties of describing suicide statistics in an international environment: The example of Luxembourg

**DOI:** 10.1192/j.eurpsy.2025.2349

**Published:** 2025-08-26

**Authors:** A. Brezina, J. Lopez-Castroman

**Affiliations:** 1 Centre Hospitalier de Luxembourg, Luxembourg City, Luxembourg; 2 Department of Psychiatry, University of Santiago de Compostela, A Coruña, Spain

## Abstract

**Introduction:**

Suicide is a major public health issue and different metrics have been put into place in order to compare suicide rates and adapt prevention strategies to local contexts. Two numbers often cited are the number of suicides in a given country in a given year and the rate of suicides per 100.000 people in this country.

As straightforward as those two indicators may seem, they may nevertheless be subject to considerable caveats in specific national contexts. As an example, we will outline the case of Luxembourg.

Luxembourg is a high-income European Union member state and benefits greatly of its multilinguality and close ties to its neighboring countries. Although other European countries also have considerable populations of foreign nationals working in, but not residing in their country, the Luxembourgish context is unique for its proportions: For a population shy of 680.000, there are about 200.000 foreign workers that cross the boarder every day.

Those frontalier-workers, as they are called, might however die by suicide in both their country of residence as well as in Luxembourg. Moreover, the Luxembourgish health care system offers considerable opportunities for patients to be treated in neighboring countries.

Those patients also might die by suicide abroad, thus not being counted into the Luxembourgish statistics.

**Objectives:**

To investigate the influence of a considerable foreign commuters demographic as well as of treatment of Luxembourgish nationals in boardering countries on the number of suicides reported for Luxembourg and its suicide rate.

**Methods:**

Analysis of data by the Ministery of Health (Ministère de la Santé et de la Sécurité sociale), the National Statistics Insitute (Institut national de la statistique et des études économiques du Grand-Duché de Luxembourg) as well as the National Health Fund (Caisse Nationale de Santé)

**Results:**

Different figures are reported for suicide cases, which cannot easily be converted into one another:

One counts the number of suicides taking place in the territory of Luxembourg, an other one describes the causes of death for Luxembourgers who died outside of the country.

Although a considerable demographic, no data exists on the proportion of frontalier-workers among suicide victims in Luxembourg.

**Conclusions:**

The study of suicide rates in Luxembourg highlights how a seemingly simple metric can prompt researchers to reconsider what exactly they aim to measure, enabling them to better design targeted prevention strategies for groups at higher risk.

**Disclosure of Interest:**

None Declared

